# Rural–urban variation in COVID-19 vaccination uptake in Aotearoa New Zealand: Examining the national roll-out

**DOI:** 10.1017/S0950268823001978

**Published:** 2024-01-04

**Authors:** Talis Liepins, Gabrielle Davie, Rory Miller, Jesse Whitehead, Brandon De Graaf, Lynne Clay, Sue Crengle, Garry Nixon

**Affiliations:** 1 Department of General Practice and Rural Health, University of Otago, Dunedin, New Zealand; 2 Department of Preventive and Social Medicine, University of Otago, Dunedin, New Zealand; 3 Te Ngira Institute for Population Research, University of Waikato, Hamilton, New Zealand; 4 Ngāi Tahu Māori Health Research Unit, University of Otago, Dunedin, New Zealand

**Keywords:** COVID-19, vaccination, rural, equity, New Zealand

## Abstract

This study aimed to understand rural–urban differences in the uptake of COVID-19 vaccinations during the peak period of the national vaccination roll-out in Aotearoa New Zealand (NZ). Using a linked national dataset of health service users aged 12+ years and COVID-19 immunization records, age-standardized rates of vaccination uptake were calculated at fortnightly intervals, between June and December 2021, by rurality, ethnicity, and region. Rate ratios were calculated for each rurality category with the most urban areas (U1) used as the reference. Overall, rural vaccination rates lagged behind urban rates, despite early rapid rural uptake. By December 2021, a rural–urban gradient developed, with age-standardized coverage for R3 areas (most rural) at 77%, R2 81%, R1 83%, U2 85%, and U1 (most urban) 89%. Age-based assessments illustrate the rural–urban vaccination uptake gap was widest for those aged 12–44 years, with older people (65+) having broadly consistent levels of uptake regardless of rurality. Variations from national trends are observable by ethnicity. Early in the roll-out, Indigenous Māori residing in R3 areas had a higher uptake than Māori in U1, and Pacific peoples in R1 had a higher uptake than those in U1. The extent of differences in rural–urban vaccine uptake also varied by region.

## Introduction

Rural–urban differences in COVID-19 vaccination uptake have been well documented, with patterns of lower vaccination rates in rural areas [1, 2]. Aotearoa New Zealand’s (NZ) vaccination coverage has been well examined from a national perspective, including comparisons by ethnicity and different health administrative districts (district health boards, DHDs) [3, 4]. However, to date, there have not been any comprehensive rural–urban analyses in NZ.

The NZ devolved responsibility for the localized roll-out of the COVID-19 vaccination programme to the then twenty DHBs. DHBs were state entities responsible for funding and providing health services to defined geographic catchments. During national health reforms in 2022, DHBs were replaced by dual agencies, Te Whatu Ora (Health New Zealand) and Te Aka Whai Ora (Māori Health Authority), spanning four amalgamated geographic and administrative regions.

The vaccine roll-out approach made DHBs responsible for key decisions surrounding the delivery of vaccinations (such as prioritizing centralized large clinics or devolving activity to community providers), the level of partnership with Indigenous Māori health providers and communities, and how specific vaccination services were established in different locations. Oversight of the national programme was managed by Manatū Hauora, the NZ Ministry of Health.

Early in the roll-out, access to vaccinations was restricted – prioritizing border and quarantine facility workers, frontline healthcare workers, those over 65 years of age, and those with ‘relevant’ underlying health conditions [5]. This was followed by limiting access by age groups, with restrictions for those aged 12+ removed in August 2021 [6].

NZ’s socio-demographic profile varies across the rural–urban spectrum and by ethnicity. Rural residents make up 19% of NZ’s total population and tend to be older, have higher levels of socio-economic deprivation, and have higher mortality rates than urban populations [7, 8]. These factors are amplified for Māori, who are also proportionately more likely to live in rural locations [8]. Furthermore, Māori face additional disadvantages of having reduced access to the social determinants of health, experiencing racism in the health system, and culturally unsuitable models of care [9–12].

Internationally, differences in access, uptake, and vaccine hesitancy have been observed by race and ethnicity [13, 14], as have reports of access challenges preventing uptake [15]. A NZ-based assessment, undertaken early in the vaccination roll-out, observed lower spatial access to vaccination services for rural populations, older people, as well as for Māori and Pacific peoples [16]. Pacific peoples are people who were born on, or have ancestral connections to, islands of the Pacific Ocean.

In this context, this study aims to quantitatively assess rural–urban differences in the uptake of COVID-19 vaccinations in NZ: nationally, regionally, and by age and ethnicity, and evaluate how this changed over time.

The study is part of a broader mixed-methods project funded by Te Whatu Ora and was conducted in parallel with a qualitative study examining localized experiences in providing vaccination services. Together, these studies aim to assist in understanding how effective the roll-out was for different populations and to guide future policy decisions around vaccination roll-outs with the ultimate aim of ensuring equitable vaccination programmes, coverage, and population protection in the future.

## Methods

The assessment period of this observational study includes the 15 fortnights from 1 June 2021 to 27 December 2021. These dates correspond to the period of greatest vaccination activity and the period of the roll-out when the vaccination was available to the general public, after the lifting of profession-based vaccine eligibility criteria [5]. ‘Fortnight’ 0 covers the period prior to 1 June 2021; fortnight 1 represents 1 June to 14 June 2021, and so on, with fortnight 15 representing 14 December to 27 December 2021 inclusive.

Data were provided by the NZ Ministry of Health. The Health Service User (HSU) 2021 dataset was used to identify the study population. The HSU dataset includes every individual who used public health services within a defined period or was enrolled in a primary care organization [17]. The HSU dataset was extracted on 1 August 2022 and included health service users and enrollees listed in the period 1 January 2021 to 31 December 2021, reflecting the study period as closely as possible. The HSU dataset included demographic variables (date of birth, sex, and ethnicity) and the meshblock (smallest geographic unit available, usually containing 30–60 dwellings [18]) of residential location.

Age of each individual was derived as at the start of the study period (1 June 2021). Children aged under 12 years were excluded to correspond to the age-based restriction in the study period and to achieve static age bands across the study period. People that died before the end of the study period were similarly excluded from the analysis, to achieve static age bands.

Immunization event information was nationally collected on the ‘COVID Immunization Register’ (CIR), a system established specifically to record individual COVID-19 vaccination events [19]. These vaccination events were linked to the HSU dataset via an encrypted unique patient identifier.

People in NZ can identify with multiple ethnicities in health administrative datasets. Ministry of Health protocols were used to derive a single ethnic group per person, based on the following order of prioritization and groupings: Māori, Pacific, and Non-Māori Non-Pacific (NMNP) [20].

Each person’s residential address meshblock was used to derive both their statistical area 2 (SA2) and DHB of domicile (an SA2 is a geographic area comprised of meshblocks and contains approximately 2,000–4,000 people in urban areas and 1,000–3,000 people in rural areas [18]). This was achieved using a concordance file [21]. Health regions were also assigned to individuals for the reporting of regional results (these four regions were not formal entities used to plan or deliver vaccination roll-out; however, they have been included in this analysis to support inter-district planning and because they comprise the current official health planning regions).

Using SA2 of domicile, each individual was then assigned a rural–urban category using the Geographic Classification for Health (GCH) [22]. The GCH has two urban (U1 and U2) and three rural (R1, R2, and R3) categories, with U1 being the ‘most urban’ and R3 the ‘most rural’. Each urban and rural category is defined based on population size and drive time thresholds that make sense in the NZ health context through qualitative and quantitative validation [7]. The GCH and corresponding population sizes are presented in Table 1.

**Table 1.** New Zealand health service user (HSU) population aged 12+ years by rurality (GCH; 5-level)

					
	HSU 2021^b^		U1		Geographic Classification for Health^a^							
	N	col %	N	row %	U2		R1		R2		R3	
Overall	4,281,980	100.0	2,731,884	63.8	756,229	17.7	509,453	11.9	236,732	5.5	47,682	1.1
Age (years)												
12–24	841,972	19.7	561,728	66.7	145,044	17.2	87,709	10.4	39,642	4.7	7,849	0.9
25–44	1,423,856	33.3	978,323	68.7	223,777	15.7	144,118	10.1	64,836	4.6	12,802	0.9
45–64	1,245,231	29.1	764,526	61.4	226,490	18.2	161,803	13.0	75,927	6.1	16,485	1.3
65+	770,921	18.0	427,307	55.4	160,918	20.9	115,823	15.0	56,327	7.3	10,546	1.4
Sex												
Female	2,166,069	50.6	1,384,413	63.9	385,860	17.8	255,393	11.8	117,452	5.4	22,951	1.1
Male	2,110,876	49.3	1,343,871	63.7	369,649	17.5	253,560	12.0	119,105	5.6	24,691	1.2
Other/Unknown	5,035	0.1	3,600	71.5	720	14.3	500	9.9	175	3.5	40	0.8
Prioritized ethnicity												
Māori	581,078	13.6	284,820	49.0	150,462	25.9	79,691	13.7	51,914	8.9	14,191	2.4
Pacific	301,492	7.0	262,724	87.1	22,233	7.4	12,450	4.1	3,463	1.1	622	0.2
Non-Māori Non-Pacific	3,399,410	79.4	2,184,340	64.3	583,534	17.2	417,312	12.3	181,355	5.3	32,869	1.0
Health Region												
Northern	1,635,271	38.2	1,414,523	86.5	74,365	4.5	74,095	4.5	54,981	3.4	17,307	1.1
Te Manawa Taki	821,236	19.2	405,047	49.3	186,643	22.7	148,115	18.0	72,803	8.9	8,628	1.1
Central	810,419	18.9	367,684	45.4	301,701	37.2	112,889	13.9	24,510	3.0	3,635	0.4
Te Waipounamu	1,015,054	23.7	544,630	53.7	193,520	19.1	174,354	17.2	84,438	8.3	18,112	1.8

a U1 is the most urban, while R3 is the most rural.

b The HSU 2021 was restricted to those aged 12+ as on 1 June 2021 and alive on 28 December 2021 that had a valid (non-missing) meshblock that mapped to the GCH.

Analysis focused on the uptake of the second primary vaccination dose, since this was the policy objective of the Ministry of Health in the study period and was considered to reflect ‘full vaccination’ status (i.e., single doses are only partial vaccination) [5]. Results of ‘vaccination uptake’ and ‘population coverage’ accordingly refer to receipt of two primary doses.

Overall age-standardized vaccination rates for each GCH category were estimated with the 2018 NZ Census Usual Resident Population (URP) used as the standard population. For ethnicity-specific age-standardized rates, the 2018 Census Māori URP was used as the standard population. Age groups used in the standardization were 12–24, 25–34, 35–44, 45–54, 55–64, 65–74, 75–84, and ‘85 & over’. Since Census population estimates are provided in 5-year age groups, three-fifths of the 10- to 14-year age group total was used to estimate the number of 12- to 14-year-olds. This was combined with the estimates for 15- to 19-year-olds and 20- to 24-year-olds to provide the count used for the youngest age group (12–24).

For each fortnight, age-standardized incident rate ratios (IRRs) were calculated for each GCH category with U1 as the reference group. This was repeated for different ethnicities, age groups, and health region groupings.

Stata SE version 17.0 was used for data management and analysis [23].

Lastly, to support the analysis of results, and wider engagement, communication, and dissemination of findings, a web-based application was developed using the R-Shiny framework [24]. This tool, publicly available at https://gch-nz.shinyapps.io/covid_vaccine/, allows users to examine COVID-19 vaccination uptake data by combinations of GCH, ethnicity, age group, and all former 20 DHB districts. The tool also includes an interactive choropleth map used to illustrate changes in vaccination rates over time for each SA2 and let users undertake bespoke local or regional analyses.

## Results

The HSU-CIR dataset contained 4,486,122 people. Individuals that died prior to the end of the study period were excluded (n = 34,334) as were those less than 12 years of age at the start of the study period (n = 40,915). Individuals who had missing residential meshblocks (n = 128,816) or whose recorded meshblock did not match a GCH category were also excluded (n = 77). The resulting dataset, summarized in Table 1, contained data for 4,281,980 individuals, 95.4% of the initial dataset.

The number of people already fully vaccinated at the starting fortnight of 1 June 2021 was 227,207 (5.3% of the total). By the end of the assessment period (28 December 2021), 3,721,831 (86.9%) people were fully vaccinated.

### Assessment of population uptake over time

**Figure 1.**
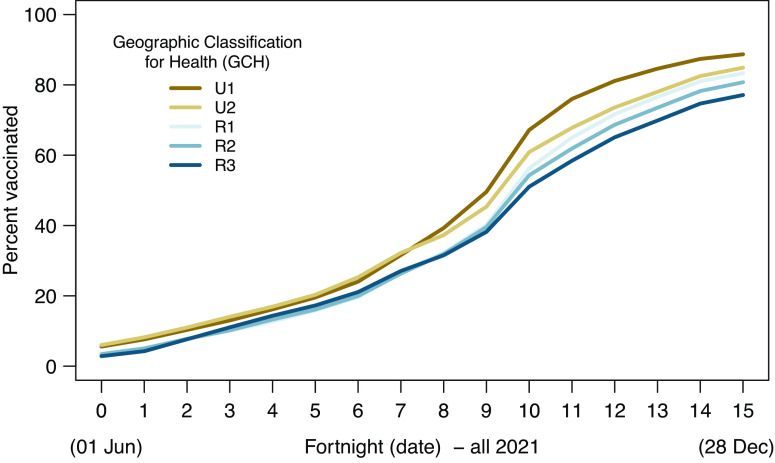
National vaccination uptake (Dose 2) by fortnight and GCH rurality category, 1 June 2021 to 27 December 2021.

Age-standardized vaccination rates for the total national population, by GCH rurality category, are shown in Figure 1.

Population uptake in each GCH rurality category follows the same general trend of incremental increases, with accelerated uptake from fortnight 9 onwards. Prior to fortnight 10, uptake did not follow a clear urban–rural gradient, with U2 and R3 areas, respectively, experiencing higher uptake than U1 and R2. However, a rural–urban gradient in uptake emerges from fortnight 10, with overall vaccination uptake lower in more rural areas of NZ.

**Figure 2.**
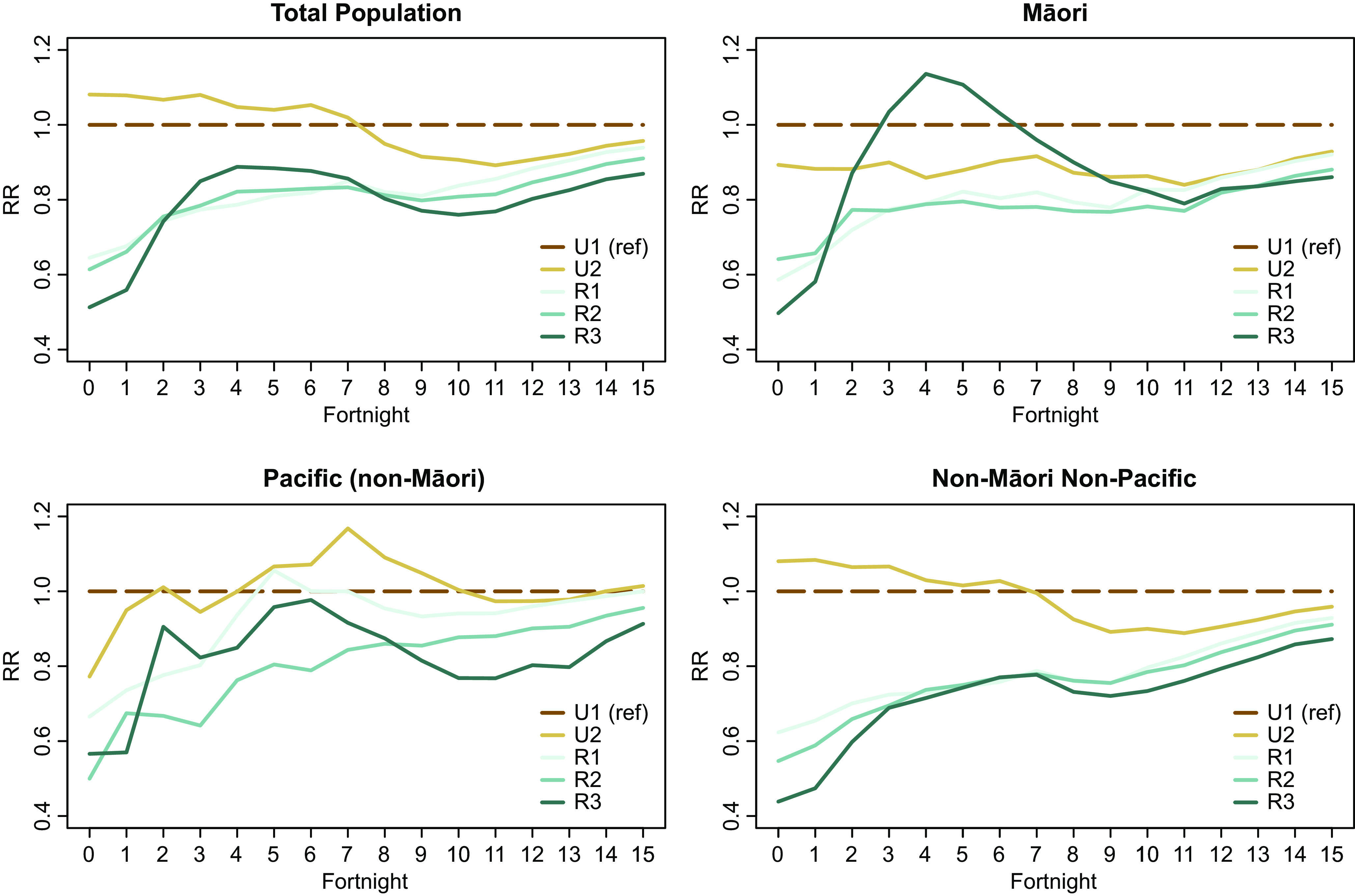
Age-standardized vaccination uptake incident rate ratios at the national level by GCH and ethnicity.

Figure 2 shows rural–urban differences in vaccination uptake using IRRs whereby the age-standardized rate of uptake in each GCH category is shown as a ratio to the level of uptake in the most urban (U1) areas. Using IRRs aids in understanding the comparative rates of uptake for each GCH category and how these vary over time.

IRRs are displayed for the total population, as well as specifically for Māori, Pacific, and Non-Māori Non-Pacific population groups. In all Figure 2 panels, the reference is the relevant U1 population group.

At the national level, overall vaccination uptake in rural areas lagged behind the most urban (U1) areas. Very early in the study period, urban areas had proportionately far greater uptake shown by the large IRR gap across fortnights 0–1, noting however that the absolute differences shown in Figure 1 are not that large. The IRR gap for rural areas closed rapidly in the first quarter of the period. The gap between rural and urban areas then stabilized until fortnight 7, at which point it widened again for rural and large provincial (U2) populations. This indicates comparatively faster population uptake in the most urban areas (U1). From around fortnight 11, rural and U2 vaccination rates began increasing again relative to those in U1 areas, but the level of vaccination did not reach U1 rates by the end of the assessment period. Of note, U2 areas had higher vaccination uptake than U1 for the first half of the study period but concluded the assessment period behind U1.

Assessing IRRs by ethnicity also illustrates generally lower uptake rates in rural areas. However, exceptions include the rapid increase in uptake for R3 Māori, which exceeded that of U1 and U2 Māori early on, and R1 Pacific uptake briefly exceeding U1 uptake rates around fortnight 5. U2 Pacific also exceeded U1 uptake midway through the assessment period and concluded the study period with higher uptake. The vaccination uptake rates for Non-Māori Non-Pacific ethnicities follow the same broad trends as seen at the national level.

### Uptake by age bands

**Figure 3.**
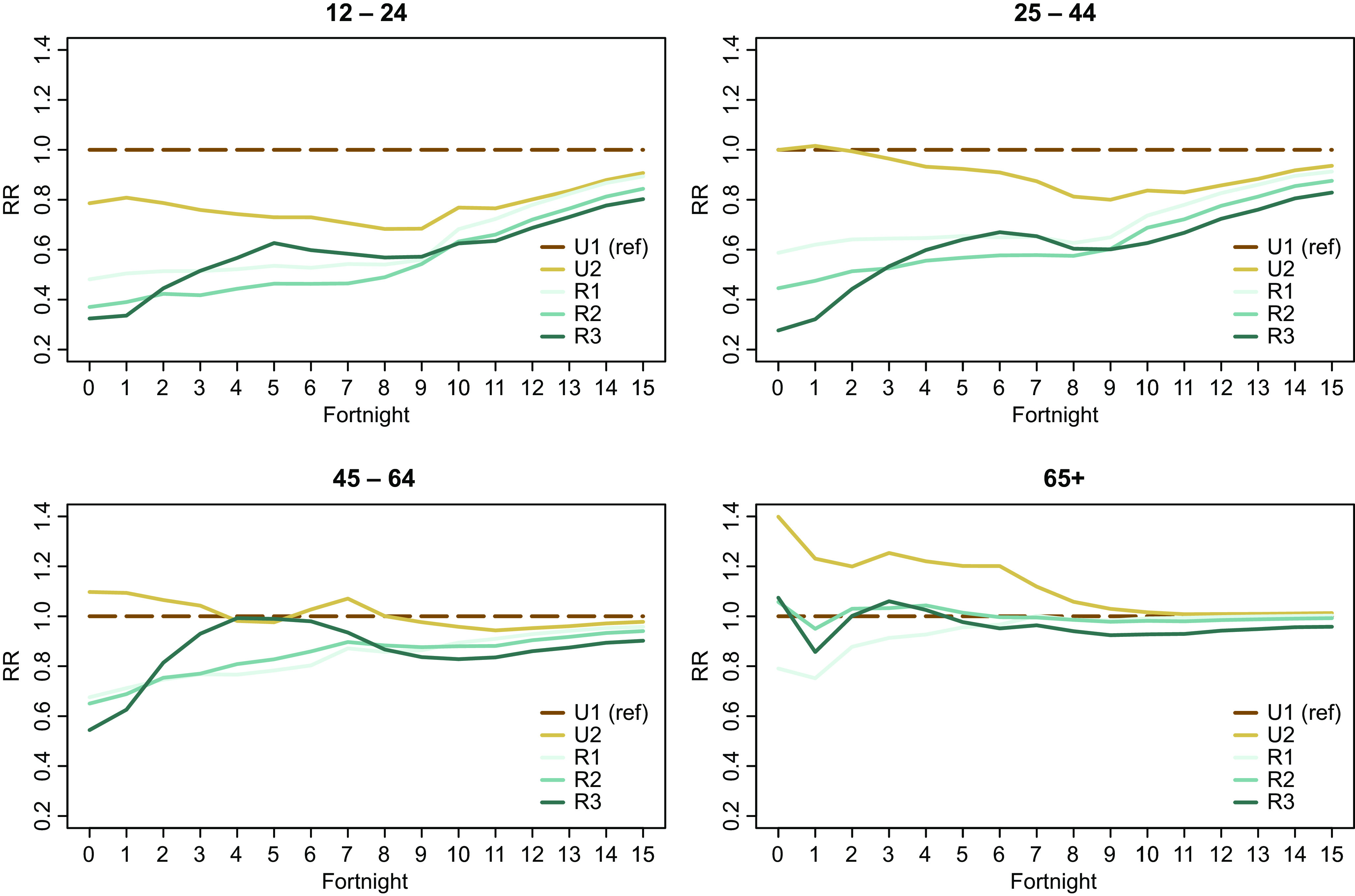
Age band vaccination uptake incident rate ratios by GCH.

Assessing uptake by age band identifies notable differences in uptake across rural and urban settings. Figure 3 displays IRRs over time for each GCH rurality category (with U1 as the reference group). Results were stratified into four age bands and displayed separately: 12–24 years; 25–44 years; 45–64 years; and 65 years and older.

For the second half of the assessment period, older people (65+) living in rural locations had broadly similar vaccination uptake rates as urban residents aged 65+. Similarly, the 45- to 64-year age group achieved relatively similar IRRs across all GCH rurality categories at the end of the assessment period (although a rural–urban gradient is still visible). In contrast, the gap between rural and urban uptake is the greatest for the age groups 12–24 and 25–44.

### Regional variations

**Figure 4.**
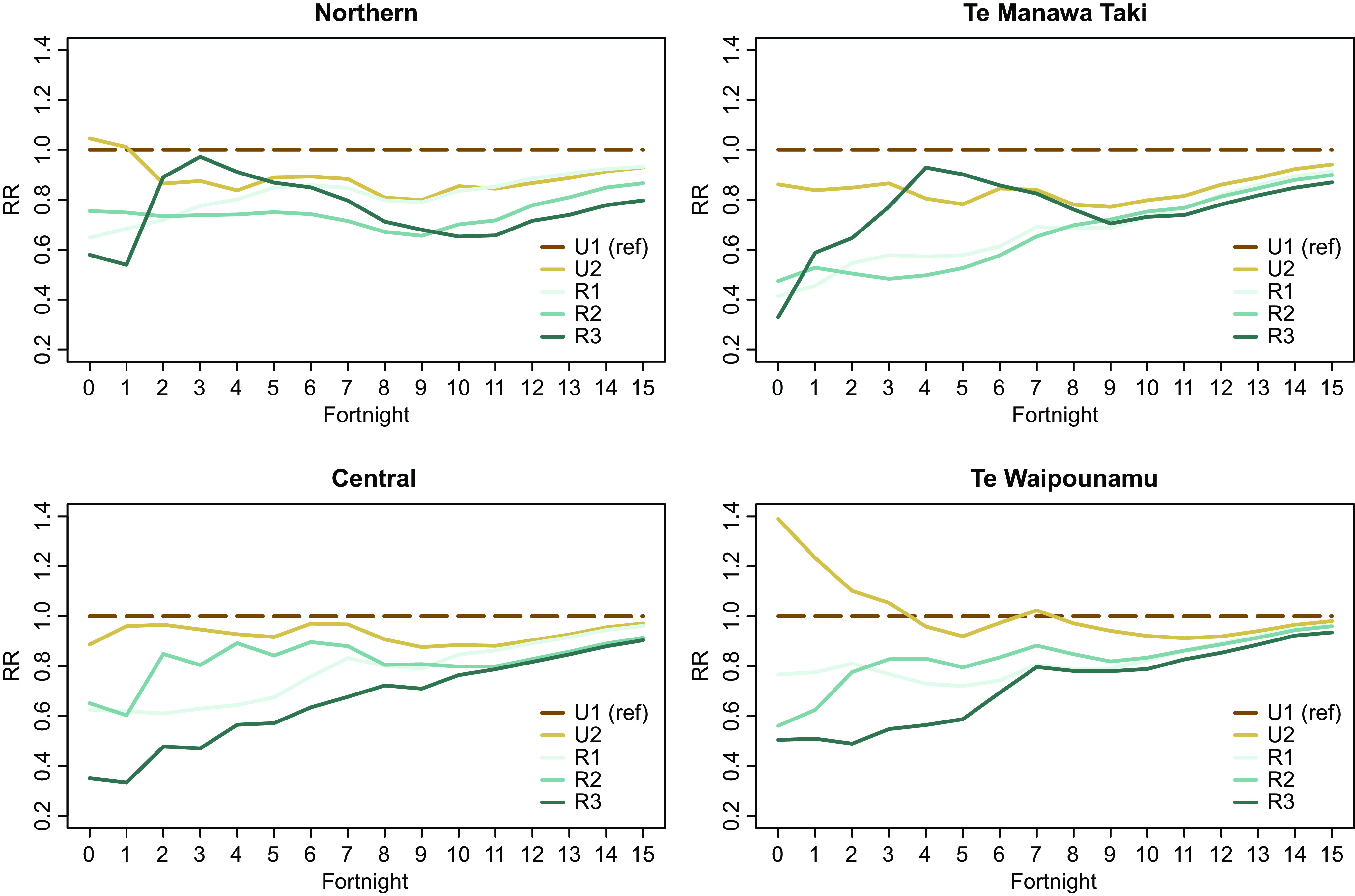
Age-standardized incident rate ratios at the national level by GCH rurality category and health region.

While broad trends are clear at the national level, regional-level snapshots also provide unique insights into population variations in uptake. Figure 4 illustrates the variation in vaccination uptake across the GCH rurality categories in NZ’s four health administrative regions.

Regional analysis again shows differences in the extent of rural–urban variation across the study period. For example, in the first six fortnights of the study period, the Northern region had lower rural–urban variation compared to other regions which had larger differences in vaccination uptake by rurality. However, by the end of the study period, Te Waipounamu had the least rural–urban variation of the four regions.

Secondly, it is apparent that there are regional differences in vaccination uptake for different GCH rurality categories. For example, vaccination uptake among R2 residents in Central and Te Waipounamu regions more closely resembled U1 uptake than it did in Northern or Te Manawa Taki. By contrast, vaccination uptake among R3 residents in these latter two regions more closely reflected U1 early in the assessment period.

## Discussion

This study identified rural–urban differences in COVID-19 vaccination uptake rates during the national roll-out’s period of peak activity. At the national level, rural uptake lagged behind urban areas, and by December 2021, it was 5–11 percentage points lower than levels of uptake in urban settings. A clear urban–rural gradient is apparent by the end of the assessment period with greater rurality associated with lower levels of vaccination uptake.

However, the rural–urban gradient in uptake is not as clear when evaluating rural–urban vaccination rates by ethnicity, age, or region.

For roughly a quarter of the period assessed, vaccination uptake for Māori in R3 exceeded Māori uptake in U1, while vaccination uptake for Pacific peoples in R1 and U2 was broadly similar to uptake for Pacific peoples in U1. The factors behind the notable uptake for R3 Māori are unable to be determined from the current data, but variations in uptake may be attributable to focused activity by rural vaccination providers early on, or differences in demand or perceptions of risk across different communities. The success in these areas was not experienced consistently for other rural areas, however – for example, Māori in R1 and both Māori and Pacific in R2 did not reach the uptake of respective U1 populations, which may reflect differences in either supply or demand.

A study undertaken during the roll-out observed spatial inequity of access to vaccination services, finding significantly lower spatial access to vaccination services for priority ethnicity groups (Māori/Pacific), rural populations, and older people [16]. This poorer access may be a contributory factor behind the observed differential vaccination rates for different rural Māori and Pacific communities, especially given previous reports that one in five Māori and Pacific experiences transport costs as a barrier to accessing primary care services [25]. Improving access to vaccination services for Māori and Pacific to aid uptake is an emphasized policy priority [26].

Vaccination uptake among older people in rural areas largely reflected urban uptake rates. However, vaccination rates of younger people in rural areas tended to be lower than those of younger people in urban areas.

The observed differences in uptake between age groups may be partially due to the general prioritization of older people in the vaccination roll-out (especially early on). However, employment, study, and/or childcare obligations combined with travel-time barriers to access vaccination providers in rural areas may contribute to the lower vaccination uptake rates among younger people in rural areas. A recent NZ study found that COVID-19 vaccine hesitancy was associated with younger ages [26]. The intersection of these findings with prior reports of spatial inequity in access in rural areas [16] may partially explain the observed difference in rural–urban vaccination rates among younger people in our study. It is possible that higher levels of vaccine hesitancy among younger people are exacerbated by travel-time and accessibility barriers, which then translates into lower rural uptake rates.

The lower rural vaccination rates observed among younger age groups align with similar recent concerning findings. Relative to their urban peers, younger rural dwellers had lower rates of utilization of key secondary care services [27], but inversely higher rates of all-cause, amenable, and injury-related mortality [28].

Our study also identified differing levels of rural–urban variation in uptake by region. By December 2021, the rural–urban uptake gradient was less pronounced in Te Waipounamu, whereas differences in uptake rates between the most remote (R3) and most urban (U1) communities were most marked in the Northern region. While further research is required to understand the drivers of these differences, they could relate to regional geography, socio-demographic differences in rural communities by region [28, 29], differing approaches to regional vaccination roll-outs, and regional differences in the accessibility of vaccination services including the intermittent nature of ‘mobile’ rural clinics [16, 30].

### Strengths and limitations

The strengths of this study include the use of a comprehensive, population-level vaccination dataset that covers all COVID-19 immunization events in NZ and the fit-for-purpose GCH to understand rural–urban differences in uptake.

The findings aid understanding of how NZ’s COVID-19 vaccination programme benefited different population groups and how community uptake of vaccinations changed over time. The study adds to the literature by considering rural–urban differences in COVID-19 vaccination uptake, not previously assessed at scale in the NZ context.

This study has some limitations, however. The study relies on accuracy in the address data supplied in the HSU dataset because this address attributes each individual and their vaccination events to a specific geographic location. Addresses were not able to be triangulated for accuracy, nor were they updated throughout the assessment period: vaccination events for populations with internal movements (such as transient or seasonal workers) are counted against the geographic area at the start of the period, not necessarily the domicile location at the time of the event.

Results by ethnicity similarly rely on accuracy within source data. Documented limitations in the HSU, including the under-counting of Māori [31], may result in under- or over-estimation of Māori outcomes depending on whether those misclassified are more or less likely to have received a vaccination than those correctly classified. Further, the use of static ages based on the age of individuals at the start of the study period (1 June 2021), while useful for conceptual simplicity, will also mean vaccinations of some individuals were counted in lower age bands (this arises for those people who would have moved up an age band during the study period, but have their vaccination counted for their age ason1 June 2021).

Finally, the restriction of vaccinations to be ‘full coverage’ (second dose rates) may also present only a partial picture of community willingness and ability to receive vaccinations. Given the described challenges in rural access, there may be differences in primary and secondary dose rates that need greater exploration. Understanding differences between first and second dose rates may support future policy by assessing whether any uptake is the issue or whether access challenges in seeking a second dose were more problematic for rural populations.

### Implications

These findings, combined with those noted from previous studies in the NZ context (spatial inequity of access during the roll-out and the noting of access barriers being an important consideration in explaining vaccine hesitancy [16, 26]), suggest opportunities for improvements in vaccination models of delivery for rural and urban communities.

While NZ’s COVID-19 vaccinations were provided free of charge, improvements in outreach models of care may be a priority policy for future vaccination efforts, especially in rural areas and for younger age groups. Similarly, the augmenting of national reporting of vaccination rates to include the measurement and communication of uptake across the rural–urban spectrum would likely be valuable to support targeted efforts and awareness of rural–urban variations.

Potential avenues for further research include:

- Understanding the reasons for reduced uptake in rural communities especially for Māori, rural younger people, and working-age individuals – including examination of differences in reasons across ethnic groups.
- Further exploration of reasons behind regional differences in uptake of vaccinations.
- Identifying and introducing programme or policy changes to overcome these challenges, including tailored options for Māori communities.
